# Apoptosis Triggering, an Important Way for Natural Products From Herbal Medicines to Treat Pancreatic Cancers

**DOI:** 10.3389/fphar.2021.796300

**Published:** 2022-02-09

**Authors:** Meiyan Li, Dandan Tang, Ting Yang, Die Qian, Runchun Xu

**Affiliations:** School of Pharmacy, Chengdu University of Traditional Chinese Medicine, Chengdu, China

**Keywords:** natural products, herbal medicine, pancreatic cancer, apoptosis, signal pathways

## Abstract

Pancreatic cancer, a poor prognosis and high morbidity and mortality cancer, is a malignant tumor occurring in pancreatic exocrine glands. Currently, surgery and gemcitabine (Gem) are commonly used to treat pancreatic cancers. However, the high recurrence rate and resistance makes the therapeutic effects still unsatisfied. Apoptosis is comprehensively recognized as one of the major ways of the programmed cell death, refers to the autonomous and orderly death process of cells in order to maintain the stability of the body's environment after receiving a certain signal or stimulation. Currently, it has also been proven to be a promising way for the treatment of pancreatic cancer. Nowadays, some active ingredients from herbal medicine have been reported to be effective for the treatment of pancreatic cancer via inducing cells apoptosis. Therefore, this article reviews the current references regarding anti pancreatic cancer effects of natural products derived from herbal medicines via triggering apoptosis, and summarizes the related potential signal pathways, including death receptors mediated apoptotic pathway, mitochondrial dependent apoptotic pathway, NF-κB mediated apoptotic pathways, MAPK mediated apoptotic pathway, ERS mediated apoptotic pathway, PI3K-Akt mediated apoptotic pathway, and other pathways such as JAK-STAT signal pathway, which can lay a certain foundation for the research and development of new natural products against pancreatic cancer.

## Introduction

Pancreatic cancer, also called “King of the cancer”, is one of the most malignant tumors of the digestive tract, and mostly of them are ductal adenocarcinoma originating from the ductal epithelium (Xu, 2019). With the progress of China's population aging and changes in residents' lifestyle and diet, the incidence of pancreatic cancer would continue to increase, becoming a major public health problem threatening human life and health. Pancreatic cancer has the characteristics of high degree of malignancy, insidious onset, and poor prognosis. Currently, similar to other cancers, surgery remains the predominant way for radical cure of pancreatic cancer, however patients undergoing surgical treatment are prone to recurrence and metastasis, which is one of the main reasons for the extremely poor prognosis of pancreatic cancer. Therefore, the chance of being controlled by surgical resection is only 10–15%. Gemcitabine (Gem) is commonly used as one of the first line drugs for the treatment of advanced pancreatic cancers (Li et al., 2014) due to its main metabolites can be incorporated into the DNA of tumor cells to activate apoptotic pathway of tumor cells. But after Gem acts on pancreatic cancer, NF-κB expression activity is abnormally increased in pancreatic cancer patients. NF-κB is an important regulator of the Bcl-2 promoter of the inhibitor of apoptosis protein. The abnormal increase in NF-κB activity leads to the activation of the Bcl-2 promoter, which ultimately leads to the acceleration of the proliferation and division of cancer cells via inhibition of apoptosis, which is an important reason for Gem resistance (Jin, 2013). Therefore, there is an urgent to develop new treatment methods and drugs for pancreatic cancer to provide better help for patients. Interestingly, current studies have shown that abundant natural effective ingredients could help induce apoptosis in various types of tumor cells, including lung cancers, pancreatic cancer cells and liver cancers, etc (Wu et al., 2015; Newman and Cragg, 2020). Compared to Gem, effective ingredients in herbal medicine are high curative effect, low toxic and resistance. Therefore, herbal medicine and ethnic medicines have become a hotspot to search for anti-cancer active ingredients. Apoptosis induction is an important mechanism of herbal medicine in the treatment of pancreatic cancer, which is a self-protection mechanism activated, expressed, and regulated by a series of specific genes. If the apoptosis signaling pathway is disordered, it will directly lead to the occurrence and progression of cancer.

Currently, much attentions have focused on the mechanism of herbal medicine inducing pancreatic cancer cell apoptosis, unfortunately the mechanisms were not clear. Therefore, the present paper aims to summarize the anticancer agents against pancreatic cancer cells derived from herbal medicines including extracts and monomers via inducing apoptosis, which would be beneficial for finding novel candidate drugs from herbal medicines for treating pancreatic cancers.

### Apoptosis and Pancreatic Cancer

Currently, the cell death modes discovered mainly include apoptosis, necrosis, autophagy, and pyroptosis, which have great differences in morphological characteristics and biochemical signal transduction. Apoptosis is established as the major mechanism of development and programmed cell death (Peng et al., 2015). In 1972, Kerr first used the term “apoptosis” to describe the unique death pattern of cells. Apoptosis is characterized by morphological changes of the cell targeted for death that include nuclear fragmentation and condensation, mitochondrial outer membrane permeabilization, membrane blebbing, cell shrinkage and apoptotic body formation. The study of different model systems has revealed the important role of apoptosis in normal development and homeostasis. Apoptosis refers to the autonomous and orderly death process of cells in order to maintain the stability of the body’s environment after receiving a certain signal or stimulation (Wang., 2018). That is to say, apoptosis is a self-protection mechanism activated, expressed, and regulated by a series of specific genes (Zhang et al., 2019). In addition, apoptosis also plays a defensive role in immune response or clearing damaged cells. Apoptosis can be used as a quality control for homeostasis (Liu et al., 2017). Pancreatic Cancer evolves through precursor lesions, known as pancreatic intraepithelial neoplasia (PanIN 1–3) to invasive ductal cancer. PanINs originate from the small pancreatic ducts, but the cell of origin of these PanINs or Pancreatic Cancer, whether ductal, acinar, or progenitor cell, remains elusive. But in the end different step-wise molecular alterations bring about the development of Pancreatic Cancer from PanINs. Other hits in the genome need to be sequentially accrued for the evolution of invasive cancer from the hyperplastic PanIN1. These include telomere shortening (PanIN1), loss of function of the tumor suppressor p16INK4a (PanIN2) and loss of function of tumor suppressors SMAD4 and TP53 (PanIN 3). These subcellular changes lead to down-regulation of apoptotic machinery (Modi, et al., 2016). Therefore, how to promote the apoptosis of pancreatic cancer cells provides a feasible direction for the development of anti-pancreatic cancer drugs.

### Natural Products From Herbal Medicines Have Potential Effects Against Pancreatic Cancer Through Apoptosis.

Apoptosis ways mainly include mitochondrial dependent apoptotic pathway, and death receptors mediated apoptotic pathway, etc. In this part, we summarized the reported natural products including extracts and monomers against pancreatic cancers via triggering apoptosis, and introduced them based on the apoptotic pathways or signal transduction ways.

### Death Receptors Mediated Apoptotic Pathway

The death receptor mediated apoptotic pathway, is mainly triggered by extracellular stimuli which are commonly recognized by the tumor necrosis factor receptor (TNFR) family of proteins, such as TNFR, Fas and TRAIL-R. Activated death receptors by their ligands, such as TNF-α, FasL, and TRAI, would further form the death inducing signaling complex, and subsequently bind to the Caspase-8, resulting in the activation of Caspase-8. Then, the activated Caspase-8 could further activate the Caspase-dependent apoptotic pathway via two routes. First, the activated Caspase-8 catalyzes Caspases (including Caspase-3 and -7) to induce apoptosis. Secondly, the activated Caspase-8 can activate the cleavage of Bid to trigger the mitochondrial dependent apoptotic pathway, promoting the release of Cytochrome C (Cyt-C) into cytoplasm from mitochondria, apoptosome formation, Caspase-cascade reaction, PARP cleavage, and ultimately leading to cell apoptosis (Scatizzi et al., 2007).

**FIGURE 1:**
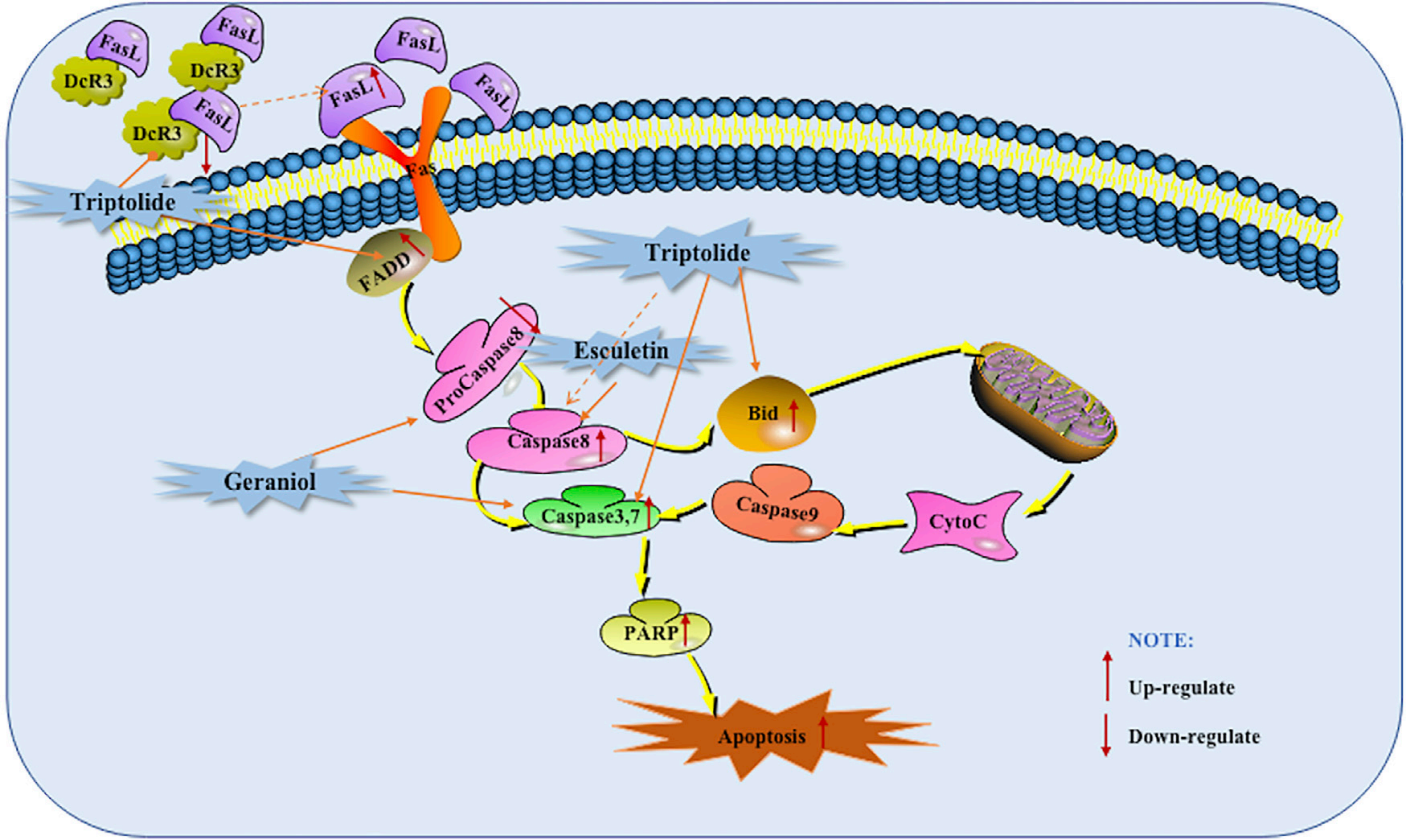
Death receptors mediated apoptotic pathway of pancreatic cancer induced by herbal medicines.

As early as 2006, Wang et al. found that the apoptosis induced by Triptolide is related to the activation of Caspase-3 and Caspase-8 and the cleavage of polymerase and Bid (Bcl-2 interacting domain death agonist) (Wang et al., 2006). DcR3 is a new member of TNFR superfamily, also known as TR6 or M68. It is a special apoptosis inhibitor that can be combined with tumor necrosis factor Fas L and negatively regulate their mediated cells (Sun et al., 2013). In 2012, Wang et al. found that Triptolide can induce apoptosis in pancreatic cancer cells by down-regulating DcR3 and up-regulating FADD, down-regulating DcR3 promoted the cleavage of Bid and Caspase-3 and the activation of Caspase-8 was also found. And further *in vivo* studies found that Triptolide decreased DcR3 levels and increased cleaved caspase-3, and found that the tumor suppressor effect of Triptolide can be significantly enhanced by DcR3 siRNA (Wang et al., 2012). In 2013, Jin found that the use of Geraniol can reduce the tumor volume in BALB/c mice, and it becomes more obvious as the number of days increases. Further *in vitro* studies found that Geraniol induced BXPC-3 cells apoptosis is related to the down-regulation of pro-Caspase-8 and pro-Caspase-3 (Jin, 2013). In addition, Arora et al., in 2016 also found that the use of Esculetin increased the cleavage of Caspase-8, which also indicates that the extrinsic apoptotic pathway is activated (Arora et al., 2016). The potential mechanisms of herbal medicine for inducing apoptosis in this part are summarized in Figure 1 and Table 1.

**TABLE 1:** Apoptosis-inducing activity of Chinese herbal medicine and its active ingredients in pancreatic cancer cells.

Potential pathways	Detail mechanisms	Extracts/Monomers	Cells/animals	Related genes/Proteins	Refs
Death receptors mediated apoptotic pathway	Activate Caspase-3 and -8; Crack Bid	Triptolide	PANC-1 cells	Caspase-3, Caspase-8; Bid	Wang et al. (2006)
	Down-regulate DcR3	Triptolide	AsPC-1, MiaPaCa-2, BxPC-3, PANC-1, SW480 cells, Athymic mice	DcR3	Wang et al. (2012)
	Up-regulating Caspase-3 and -8	Geraniol	BXPC-3, HPC-Y5 cells; BALB/c Nude mice	Caspase-8, Caspase-3	Jin. (2013)
	Increase Caspase-8	Esculetin	PANC-1, MIA PaCa-2, AsPC-1 cell lines	Caspase-8	Arora et al. (2016)
Mitochondrial dependent apoptotic pathway	Increase Bax and Caspase-3; Increase Cyt-C and AIF	Capsaicin	AsPC-1, BxPC-3 cells	Bax, Caspase-3, Cyt-C, AIF	Zhang et al. (2008)
	Up-regulating Caspase-3 and Bax	Triptolide	SW1990 cell line	Caspase-3, Bax	Zhou et al. (2008)
	Increase miR-204, Decrease Mcl-1	Triptolide	Cancer patients; S2-VP10, AsPC-1, MIA PaCa-2	miR-204, Mcl-1	Chen et al. (2013)
	Up-regulating Bim; Activate Caspase-3	Resveratrol	PANC-1, MIA PaCa-2, Hs766T, AsPC-1 cells	Bim, Caspase-3	Roy et al. (2011)
	Down-regulate Mcl-1; Up-regulating Puma, Bim	Resveratrol	PANC-1, BxPC-3 cells	Mcl-1, Puma, Bim	Duan et al. (2016)
	Decrease Bcl-2/Bax	Casticin	PANC-1, BxPC3, HPAC, COS-7, FRT cells	Bcl-2, Bax	Ding., (2012)
	Up-regulating ROS; Decrease Bcl-2/Bax; Up-regulating Cyt-C; Activate Caspase-3	Isoalantolactone	PANC-1, BxPC3, HPAC, COS-7, FRT cells	Bcl-2, Bax, Cyt-C, Caspase-3	Ding., (2012)
	Down-regulate Bcl-2 and Bcl-xl; Up-regulating Bax; Activate Caspase-3	Proanthocyanidins	Female athymic nude mice; Miapaca-2, PANC-1, AsPC-1 cells	Bcl-2, Bcl-xl, Bax, Caspase-3	Prasad et al. (2012)
	Decrease Bcl-2/Bax; Activate Caspase-3	Oridonin	PANC-1 cells	Bcl-2, Bax, Caspase-3	Qi et al. (2012)
	Decrease MCMP (Δ ψ m); Down-regulate Bcl-2; Up-regulating caspase-3 and -9	Brucein D	Capan-2 cells	Bcl-2, Caspase-9; Caspase-3	Liu et al. (2012)
	Increase Cyt-C; Activate Caspase pathway	Geraniol	BXPC-3, HPC-Y5 cells; BALB/c Nude mice	Cyt-C	Jin., (2013)
	Up-regulating Bax, Bad, Puma; BIM, PARP, Caspase-7; Down-regulating Bcl-2, Bcl-XL	Terphenyllin	PANC-1, AsPC-1, CFPAC1, HPNE, SW 1990, Capan-2, HPAC cells	Bax, Bad, Puma, Bim, PARP, Caspase-7, Bcl-2, Bcl-x1, Bcl-2, Ser70, Caspase-7	Zhang.et al. (2020)
	Up-regulating Caspase-3 and -9, Bak; Down-regulating Bcl-XL; Increase; Decrease MCMP (Δ ψ m); Bcl-XL, Bak	Quercetin	PANC-1 cells	Caspase-9, Caspase-3	Lee et al. (2013)
	Up-regulating Bak, Caspase-3 &-9; Down-regulating Bcl-2, Bcl-XL; Increase Cyt-C	Bitter gourd juice	BxPC-3, MiaPaCa-2, AsPC-1, Capan-2 cells; BALB/c Nude mice	Bak, Bcl-2, Bcl-XL, Cyt-C, Caspase-3, Caspase-9	Kaur et al. (2013)
	Down-regulating Bcl-2, survivin; Up-regulating Bax, Caspase-3and -9	Ginsenoside Rh2	BxpC-3 cells	Bcl-2, Survivin, Bax, Caspase-3, Caspase-9	Tang et al. (2013)
	Activate Caspase-3; Up-regulating Bax; Down-regulating Bcl-2; Bcl-XL, c-Myc, Caspase-3	Longikaurin E	PANC1, AsPC-1, BxPC-3 cells	Bax, Bcl-2	Cheng et al. (2015)
	Up-regulating Bax; Down-regulating Bcl-2	YCHD	PANC-1 cells	Bax, Bcl-2	Zhou et al. (2015)
	Increase ROS; Up-regulating Bax, Caspase-3; Down-regulating Bcl-2	Nimbolide	hTERT HPNE, HPAC、PANC-1, MIAPaCa-2; BALB/c Nude mice	Bax, Caspase-3, PARP, Bcl-2	Subramani et al. (2016)
	Up-regulating Bax, Caspase-3 and -9; Down-regulating Bcl-2	Aconitine	Miacapa-2, PANC-1	Bax, Caspase-3, Caspase-9, PARP1, Bcl-2	Ji et al. (2016)
	Increase Cyt-C; Up-regulating Caspase-3 and -9	Esculetin	PANC-1, MIA PaCa-2, AsPC-1 cell lines	Cyt-C, Caspase-3, Caspase-9	Arora et al. (2016)
	Up-regulating p53 and Bax; Down-regulating Bcl-2	Ethylacetate fraction	PANC-1 cells	p53, Bax, Bcl-2	Ding et al. (2017)
	Up-regulating HTRA3, Bax	Paeoniflorin	Capan-1, MIAPaCa-2 cell lines	HTRA3, Bax	Li et al. (2017)
	Increase ROS; Decrease MCMP (Δ ψ m); Up-regulating Bax; Down-regulating Bcl-2	Glychionide-A	PANC-1, hTRET-HPNE cells	Bax, Bcl-2	Yu et al. (2019)
	Up-regulating Caspase-3, Bax; Down-regulating Bcl-2	Piperine	PANC-1 cells	Caspase-3, Bax, Bcl-2	Zhong et al. (2020)
	Increase ROS, Cyt-C; Up-regulating Caspase-3 and -9	Tephrosin	A549, MCF-7, HepG2; SHG-44, CFPAC-1, MIAPaCa, PANC-1, SW1990 cell lines; BALB/c Nude mice	Cyt-C, Caspase-3, Caspase-9, PARP	Du et al. (2021)
	Up-regulating Bax and Caspase-3; Down-regulating Bcl-2	Icariin	BxPC-3 cells	Bax, Bcl-2, Caspase-3	Huang et al. (2021)
	Up-regulating Bax; Down-regulating Bcl-2, Wnt/β-catenin	Schisandrin B	PANC-1 cells	Bax, Bcl-2, Wnt4, Wnt5a, β-catenin	Wang et al. (2021a)
ERS mediated apoptotic pathway	Up-regulating GRP78, eIF2α, ATF4, GADD153	capsaicin	PANC-1, SW1990 cells; BALB/c Nude mice	eIF2 α, ATF4, GADD153	Lin et al. (2013)
	Up-regulating CHOP	Bitter gourd juice	BxPC-3, MiaPaCa-2, AsPC-1, Capan-2 cells; BALB/c Nude mice	CHOP	Kaur et al. (2013)
	Up-regulating phosphorylation-PERK, Grp78/BiP, GADD153/CHOP	Quercetin	PANC-1 cells	GADD153, CHOP	Lee et al. (2013)
PI3K-Akt mediated apoptotic pathway	Down-regulating Tyr458, Ser473; Up-regulating Caspase-3	Capsaicin	PANC-1 cells; BALB/c Nude mice	Tyr458, Ser473	Zhang et al. (2013)
	Down-regulating PI3K/AKT signaling	Longikaurin E	PANC1, AsPC-1, BxPC-3 cells	PI3K, Akt	Cheng et al. (2015)
	Down-regulating PI3K/AKT signaling	Brucein D	PANC-1, Capan-1, Capan-2, GES-1, SW-1990 cells; BALB/c Nude mice	PI3K, Akt	Lai et al. (2017)
	Down-regulating PI3K/AKT signaling	Timosaponin-AIII	PANC-1, BxPC-3 cells	PI3K, Akt	MarElia et al. (2018)
	Down-regulating Akt/mTOR signaling	Kaempferol	PANC-1, Mia PaCa-2 cells; BALB/C Nude mice	Akt, mTOR	Wang et al. (2021b)
NF-κB Mediated Apoptotic Pathways	Inhibit NF-κB, p65 activation; Decrease transcription of Bcl-2 and XIAP	Brucetin D	PANC-1 cells; BALB/c Nude mice	NF-κB	Lau et al. (2010)
	Down-regulating Bcl-2, Livin and Survivin; Up-regulating Bax	Geraniol	BXPC-3, HPC-Y5 cells; BALB/c Nude mice	Livin, Survivin, Bcl-2, Bax	Jin.,2013
	Inhibit Nrf2 phosphorylation, NF-κB	Esculetin	PANC-1, MIA PaCa-2, AsPC-1 cell lines	Nrf2, NF-κB	Arora et al. (2016)
	Down-regulating NF-κB, cIAP1, cIAP2, survivin, Bcl-2/Bax; Up-regulating Caspase-3	Lycopene	PANC-1 cells	NF-ΚB, cIAP1, cIAP2, Caspase-3, Bcl-2, Bax	Jeong et al. (2019)
	Inhibit NF-κB; Up-regulating Caspase-3	Sinomenine	Capan-1 cells	NF-κB, Caspase-3	Chen et al. (2020)
MAPK Mediated Apoptotic Pathway	Down-regulating ERK_1/2_	Curcumin	H1299, PANC-1, p34, PC-14 cells	ERK_1/2_	Lev-Ari et al. (2006)
	Increase ROS; Up-regulating p38	Brucetin D	PANC-1 cells; BALB/c Nude mice	p38	Lau et al. (2010)
	Activate p38	Bitter gourd juice	BxPC-3, MiaPaCa-2, AsPC-1, Capan-2 cells; BALB/c Nude mice	p38, ERK_1/2_	Kaur et al. (2013)
	Activate JNK and p38; Inhibit ERK	Phycocyanin	PANC-1, Capan-1, BxPC3, H460, QSG-7701, AC-16, HepG2, BGC-823, DU145, MCF-7, HK-2 cells	JNK, p38, ERK	Liao et al. (2016)
Others	Increase miR-7; Decrease SET8	Curcumin	AsPC-1, BxPC-3 cells	miR-7	Ma et al. (2014)
	Up-regulating miR-340; Down-regulating XIAP	Curcumin	PANC-1, HEK293 cells	miR-340, XIAP	Yang et al. (2017)
	Up-regulating miR-101, Caspase-3; Down-regulating Mcl-1	Honokiol	PANC-1, SW1990 cells; BALB/c Nude mice	miR-101, Caspase-3, Mcl-1	Wang et al. (2020)
	Up-regulating miR-9	Icariin	BxPC-3 cells	miR-9	Huang et al. (2021)
	Inhibit Hsp70, 5-LOX	Triptolide	PANC-1, MiaPaCa-2, SW-1990 cells; Female nude mice	Hsp70, 5-LOX	Phillips et al., 2007
					Zhou et al. (2007)
	Down-regulation of EZH2, Trx; Activate ASK1	Capsaicin	AsPC-1, BxPC-3 cells; BALB/c Nude mice	Trx, ASK1	Pramanik and Srivastava, (2012)
		Diosgenin	Patu8988 and Panc-1, SW1990 cell line, Nude mice	EZH2	Guo et al. (2019)
	Inhibit IL-6/JAK2/STAT3 signaling	Gentiopicroside	PANC-1 cells	IL-6, JAK2, STAT3	Meng et al. (2020)
	Down-regulating PD-L1	Polyphyllin VII	PANC-1, Miapaca-2cells	PD-L1	He et al., 2021; Hu et al., 2020

YCHD, Yin Chen Hao Decoction; Cyt-C, cytochrome C; AIF, apoptosis inducing fact.

### Mitochondrial Dependent Apoptotic Pathway

Mitochondria are the energy factories for cell activities, and the integrity of the structure and function of mitochondria is an important prerequisite to ensure the normal life activities of cells. Mitochondria mediated cell apoptosis is also known as the endogenous pathway of cell apoptosis, When the molecules separating the outer and inner mitochondrial membranes are released into the cytoplasm through the outer mitochondrial membrane permeabilization (MOMP), the Mitochondrial dependent apoptotic pathway begins. The process is controlled by anti-apoptotic proteins and pro-apoptotic proteins of Bcl-2 family.

In the Bcl-2 family, the anti-apoptotic proteins include Bcl-2, A1, Mcl-1, and the pro-apoptotic members were further divided into effector molecules, [e.g., Bak (Bcl-2 antagonist killer 1), Bax (Bcl-2 associated x protein)] and BH3-only proteins, [e.g., Bad (Bcl-2 antagonist of cell death), Bim (Bcl-2 interacting mediator of cell death), Bid (Bcl-2 interacting domain death agonist), Bik (Bcl-2 interacting killer) and Puma (p53-upregulated modulator of apoptosis)] (Chipuk and Green, 2008). If Bax and Bak are transferred from cytoplasm to mitochondrial membrane, forming transmembrane pores and decreasing the mitochondrial membrane potential (MCMP). At the same time, due to the disruption of the pro-apoptotic/anti-apoptotic protein balance, the permeability transition pore (PT) is induced to open, which further reduces the MCMP, thereby increasing the permeability of the mitochondrial membrane and promoting the release of Cyt-C. In turn, the mitochondrial respiratory chain will function abnormally and the cell will die due to lack of ATP. Cyt-C is not only involved in electron transport but also an important apoptosis initiation protein, because Cyt-C in the cytoplasm combines with APaf-1 protein and then combines with dATp/ATP to form a trimer and then oligomerizes to become apoptotic protein. The CARD domain is exposed after APaf-1 oligomerization and specifically adsorbs Caspase-9 zymogen into the apoptotic body. Caspase-9 is activated and therefore reaches a series of downstream Caspases into the apoptosis process and ultimately leads to apoptosis. (Jin., 2013; Lai et al., 2021). Nowadays, increasing scientific evidences have suggested that natural products are effective agents for the treatment of pancreatic cancer based on induction of mitochondrial dependent apoptosis.

In 2008, Zhang et al. found that Capsaicin which is an interesting alkaloid isolated from the *Capsicum annuum* promoted the apoptosis of mice tumor cells, which was related to the up-regulated of Bax and Cleaved (C)-Caspase-3 and the increased of Cyt-C and AIF in the cytoplasm. The same results were also found for AsPC-1 and BxPC-3 cells when conducting *in vitro* studies (Zhang et al., 2008). Another research by Zhou et al., in 2008 found that Triptolide induced human pancreatic cancer cells to undergo apoptosis with typically apoptotic characteristics. Further research found that caspase-3 and Bax were significantly up-regulated in SW1990 cells treated with TL (Zhou et al., 2008). Mcl-1 is a member of the Bcl-2 superfamily. Recent genome-wide research suggests that Mcl-1 is subject to increased gene copy number across more than two dozen cancer types. Exploiting drug regimens targeting pathways that down-regulate Mcl-1 expression is therefore a current strategy in cancer therapy. In 2013, the results of Chen et al. showed that Triptolide can reduce the level of Mcl-1 by increasing the expression of miR-204, eventually induce apoptosis of pancreatic cancer cells (Chen et al., 2013). In 2011, Roy et al. found that Resveratrol which is a versatile natural monomer in grape skins and red wine can induce apoptosis in PANC-1, MIA PaCa-2, HS766T and AsPC-1 cells in a dose-dependent manner. Further researched found that Resveratrol induced apoptosis is related to the regulation of Bim and the activation of Caspase-3 (Roy et al., 2011). Subsequently, Duan et al. found in 2016 that Resveratrol significantly induces the PARP and Cleaved-Caspase-3 in PANC-1 and BxPC-3 cells. Resveratrol also can promote the down-regulation of Mcl-1, the up-regulation of Puma and the Bim (Duan et al., 2016). Under normal circumstances, the pro-apoptotic protein Bax, which exists in the cytoplasm, plays a negative regulatory effect against the anti-apoptotic protein Bcl-2, that is, Bcl-2/Bax determines cell apoptosis. In 2012, when Ding screened natural active compounds, it was found that Casticin could significantly increases the expression of Bax in PANC-1 cells while inhibiting the expression of Bcl-2. That is to say, Casticin caused cell apoptosis by reducing the ratio of Bcl-2/Bax. In this experiment, it was also found that Isoalantolactone can increase the level of ROS in PANC-1 cells in a dose-dependent manner, increase the expression of p38 and Bax, and accompanied by the release of Cyt-C and the activation of Caspase-3, it supports the view that Isoalantolactone induces pancreatic cancer PANC-1 cell apoptosis through an endogenous pathway (Ding., 2012). In the same year, Prasad R et al. found that Proanthocyanidins extracted from grape seeds significantly induced apoptosis of Miapaca-2 and PANC-1 cells. The potential mechanism of Grape seed Proanthocyanidins (GSPs) induction of apoptosis is related to the decrease of Bcl-2 and Bcl-xL levels, the increase of Bax and the activation of Caspase-3. Further *in vivo* studies found that the percentage of activated caspase-3 in the pancreatic tumor xenografts in athymic nude mice treated with GSPs was higher than that of mice that did not receive GSPs. It was also found that the level of Bax was increased, and the levels of Bcl-2 and Bcl-xL were reduced (Prasad et al., 2012). Qi et al. found that the apoptosis of PANC-1 cells induced by Oridonin is mediated by the decrease of Bcl-2/Bax ratio and activation of Caspase-3. This study also showed that Oridonin nanosuspension is more effective than free oridonin on G2/M cell cycle arrest and apoptosis in human pancreatic cancer PANC-1 cell line (Qi et al., 2012). In this year, Liu et al. found that Brucetin D can reduce the mitochondrial membrane potential in cells, weaken the expression of Bcl-2 protein, enhance the expression of Caspase-9 and Caspase-3 and increase Capan-2 cells apoptosis (Liu et al., 2012). Later in 2019, Huang found that the relative expression of Bcl-2, PARP, and Caspase-3 proteins after the combination of Brucein D and Taxol was significantly reduced. However, the relative expression of C-Caspase-3 protein was significantly increased. It shows that the combination can induce Capan-2 cells apoptosis by activating the Caspase-pathway (Huang et al., 2019). In 2013, Jin et al. found that Geraniol can reduce the expression of Cyt-C in the mitochondria of pancreatic cancer cells and increase the expression of Cyt-C in the cytoplasm. In turn, it activates the Caspase-pathway in the cytoplasm to promote the apoptosis of BXPC3 cells (Jin., 2013). In the same year, Lee et al. found that Quercetin increases the activity of Caspase-9 in PANC-1 cells, and induces the activation of Caspase-3. It also causes the change of PANC-1 mitochondrial membrane potential, decreases Bcl-xL and increases expression of Bak (Lee et al., 2013). Kaur et al. studied Bitter Gourd Juice (BMJ) and found that it significantly induced the pro-apoptotic protein Bak, and promoted the decrease of anti-apoptotic proteins Bcl-2 or Bcl-xL. The cytostatic levels of BMJ apoptotic molecules (survivin and XIAP) were significantly reduced, which also resulted in the release of Cyt-C into the cytoplasm and activated both Caspase-3 and Caspase-9 (Kaur et al., 2013). Tang et al. also found that Ginsenoside Rh2 can down-regulate Bcl-2 and surviving, and up-regulate Bax, promote the cleavage of Caspase-3 and Caspase-9, and initiate the endogenous apoptotic pathway to induce BxpC-3 pancreatic cancer apoptosis (Tang et al., 2013). Longikaurin E is a substance from the *Rabdosia longitude* and has anti-proliferation and pro-apoptotic properties in a variety of cancers. In a 2015 study by Cheng et al., it was found that Longikaurin E promoted the apoptosis of PANC-1 human pancreatic cancer cells by reducing the Bcl-2/Bax ratio and activating Caspase-3 (Cheng et al., 2015). In 2016, Subramani et al. discovered Nimbolide, a phytochemical isolated from the leaves and flowers of Neem tree. The levels of ROS, Bax, C-Caspase-3 and lytic PARP were increased and the level of Bcl-2 was decreased, thus inducing cell apoptosis through the Mitochondrial Dependent Apoptotic Pathway. In pancreatic cancer xenograft models, it was found that Nimbolide induced apoptosis is related to the increase in the expression of Bax, C-Caspase-3 and lytic PARP; and the decrease in the expression of the anti-apoptotic protein Bcl-2. These findings also confirmed the *in vitro* observations. (Subramani et al., 2016). In the same year, Ji et al. found that Aconitine can induce cell apoptosis by up-regulating the expression of pro-apoptotic factors Bax, C-Caspase-3, C-Caspase-9 and lytic PARP1, and reducing anti-apoptotic Bcl-2. Importantly, NF-κB was also reduced after Aconitine treatment (Ji et al., 2016). In this year’s study, Arora et al. found that Esculetin promotes the loss of mitochondrial membrane potential, leading to the release of Cyt-C into the cytoplasm. At the same time, the activation and cleavage of Caspase-3, Caspase-9 and Caspase-8 were also observed. All in all, Esculetin can induce apoptosis through both extrinsic apoptotic and endogenous pathways (Arora et al., 2016). In 2017, Ding et al. found that the Ethylacetate fraction (EAF) can inhibit proliferation and promote apoptosis of pancreatic cancer PANC-1 cells. The mechanism may be related to the up-regulation of p53, Bax expression and down-regulation of Bcl-2. HTRA3 belongs to the highly conserved HtrA family of stress-related serine proteases. HTRA3 sensitizes lung cancer cells to etoposide and cisplatin, where it may act as an effector of mitochondrial cell death, indicating its role as a tumor suppressor. In 2017, Li et al. found that Paeoniflorin increases the expression of HTRA3 in Capan-1 cells. The overexpression of HTRA3 also detectes an increase in the level of Bax protein. Therefore, HTRA3 exerts a new pro-apoptotic effect in pancreatic cancer cells by inducing Bax (Li et al., 2017). In 2019, Yu et al. discovered Glychionide-A, a new flavonosides have been isolated from the roots of Glychirriza glabra (Li et al., 2005). Glychionide-A increases ROS and reduces the mitochondrial membrane potential level of PANC-1 pancreatic cancer cells, indicating that Glychionide-A is ROS-mediated apoptosis and autophagy. Experiments also found that the expression of Bax increased and Caspase-9 reduced the expression of Bcl-2 in PANC-1 cells (Yu et al., 2019). In 2020, Zhong et al. found that Piperine can up-regulate the expression levels of Caspase-3 and Bax mRNA in PANC-1 cells, up-regulate the expression levels of C-Caspase-3 and Bax proteins, and down-regulate the expression levels of Bcl-2 mRNA and protein. It further shows that Piperine can induce PANC-1 cell apoptosis by regulating the Caspase-3/Bax/Bcl-2 apoptotic signaling pathway (Zhong et al., 2020). In the same year, Zhang discovered in his research that the natural drug Terphenyllin increased the expression of Bax, Bad, Puma and BIM, and inhibited the expression of Bcl-2 and Bcl-xL. The decrease of the Bcl-2/Bax ratio promotes the apoptosis of pancreatic cancer cells. At the same time, the expression of P-Bcl2-Ser70, Caspase-7 and PARP gradually decreased, and the expression of Cleaved PARP and Cleaved Caspase-7 gradually increased (Zhang, 2020). Recently, Du et al. discovered that Tephrosin, a natural carotenoid isoflavone, increases the production of reactive oxygen species (ROS) in the cell, depolarizes the mitochondrial membrane potential and releases Cyt-C to promote apoptosis. Tephrosin can also promote the apoptosis of PANC-1 and SW1990 pancreatic cancer cells by enhancing the cleavage of Caspase-3, Caspase-9 and PARP. To further study the anti-tumor effects of tephrosin in vivo, PANC-1 cells were injected subcutaneously into BALB/C nude mice. The study found that high-dose tephrosin (20 mg/kg) significantly reduced tumor growth *in vivo* compared with administration of vehicle. No pathological changes were observed in various organs after H&E staining. These results all proved the anti-tumor effect and low toxicity of tephrosin (Du et al., 2021). In the same year, Huang et al. found that Icariin increased the apoptosis rate of pancreatic cancer BxPC-3 cells, the expression of apoptosis marker proteins Bax and Cleaved Caspase-3, and down-regulated the expression of apoptosis marker protein Bcl-2 (Huang et al., 2021). In the same year, Wang et al. found that Schisandrin B can significantly induce the expression of the pro-apoptotic protein Bax and inhibit the expression of the anti-apoptotic protein Bcl-2. This study also found that the induction of pancreatic cancer PANC-1 cell apoptosis may be related to the inhibition of Wnt/β-catenin signaling pathway activation (Wang F. et al., 2021).

**FIGURE 2:**
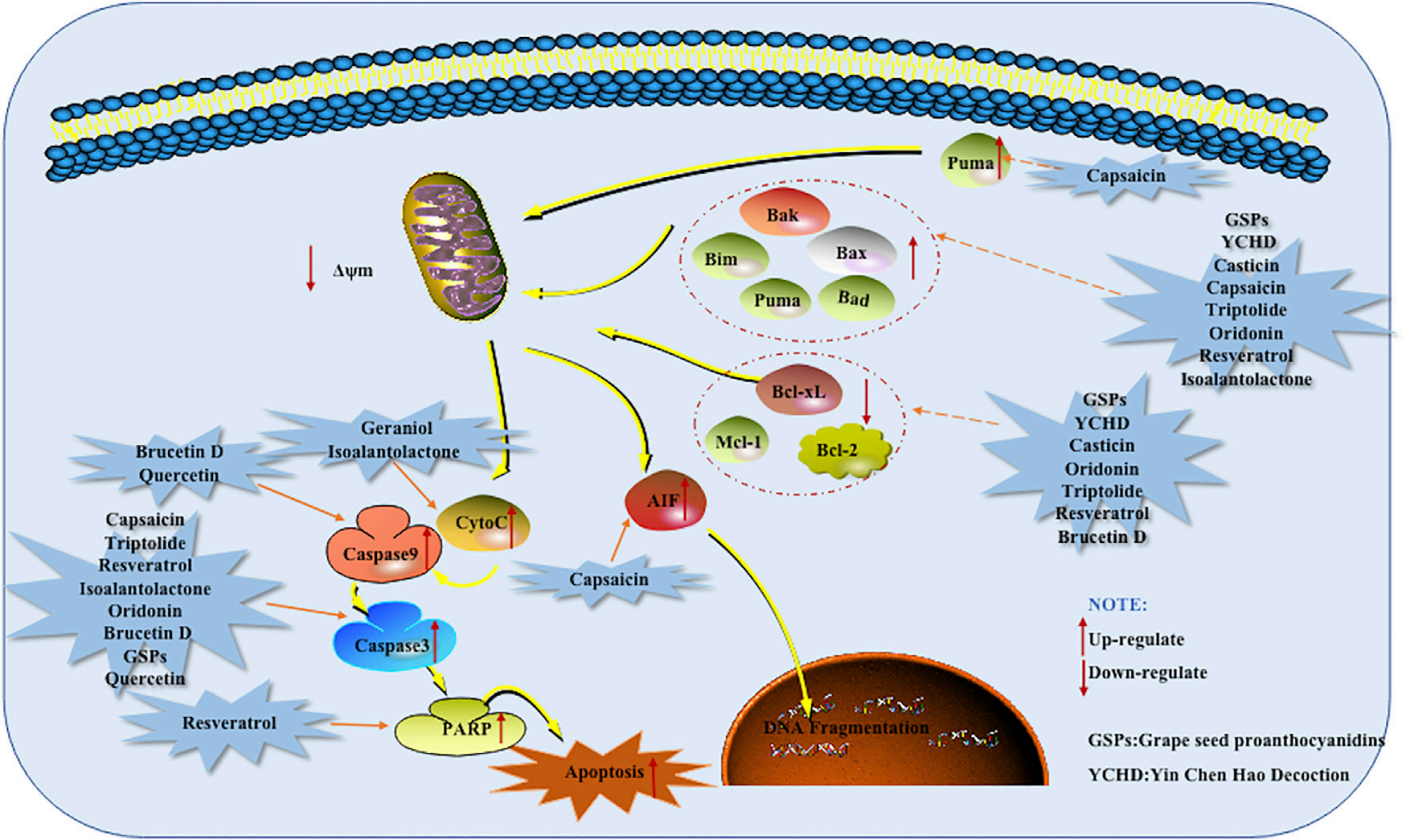
Mitochondrial dependent apoptotic pathway of pancreatic cancer induced by herbal medicines.

In addition to monomers, ancient allotment can also induce apoptosis in pancreatic cancer. Such as *Yin Chen Hao Decoction* (YCHD), which is a classic traditional Chinese medicine formula composed of three herbs: *Rheum officinale* Baill, *Artemisia capillaries* Thunb and *Gardenia iasminoides* Ellis. For a long time, YCHD can be used to treat cholestasis, hepatitis C, primary bil liver fibrosis. In addition, YCHD is an effective cancer suppressor. The anti-cancer activity of YCHD may induce cancer cell apoptosis. In 2015, Zhou et al. found that YCHD induced PANC-1 cell apoptosis, part of the mechanism was through up-regulation of Bax and down-regulation of Bcl-2 (Zhou et al., 2015). The potential mechanisms of herbal medicine for inducing apoptosis in this part are summarized in Figure 2 and Table 1.

### ERS Mediated Apoptotic Pathway

The endoplasmic reticulum (ER) is an organelle that receives various emergency stimuli and transmits relaxation signals. The endoplasmic reticulum can guide the synthesis, folding and secretion of proteins in eukaryotic cells. Endoplasmic reticulum stress (ERS) caused by endoplasmic reticulum dysfunction can enhance the folding ability of proteins, delay the translation of most proteins, and accelerate protein degradation. It is the cell’s self-protection mechanism. It can also activate the unfolded protein response (UPR), induce the expression of molecular chaperones such as GRP78, GRP94, Bip and so on, regulate the Irel/xBPT pathway, p-ERK/eIF2α pathway, up-regulate the expression of CCAAT/CHOP, and indirectly promote cell apoptosis. In addition, ESR can induce the opening of calcium channels, promote Ca^2+^ outflow, and break the BCL-2/Bax balance. These are all called ERS Mediated Apoptotic Pathway (Zhang et al., 2019). At present, there are many reports that Chinese herbal medicines and their monomers can induce pancreatic cancer cell apoptosis through the ERS Mediated Apoptotic Pathway.

**FIGURE 3:**
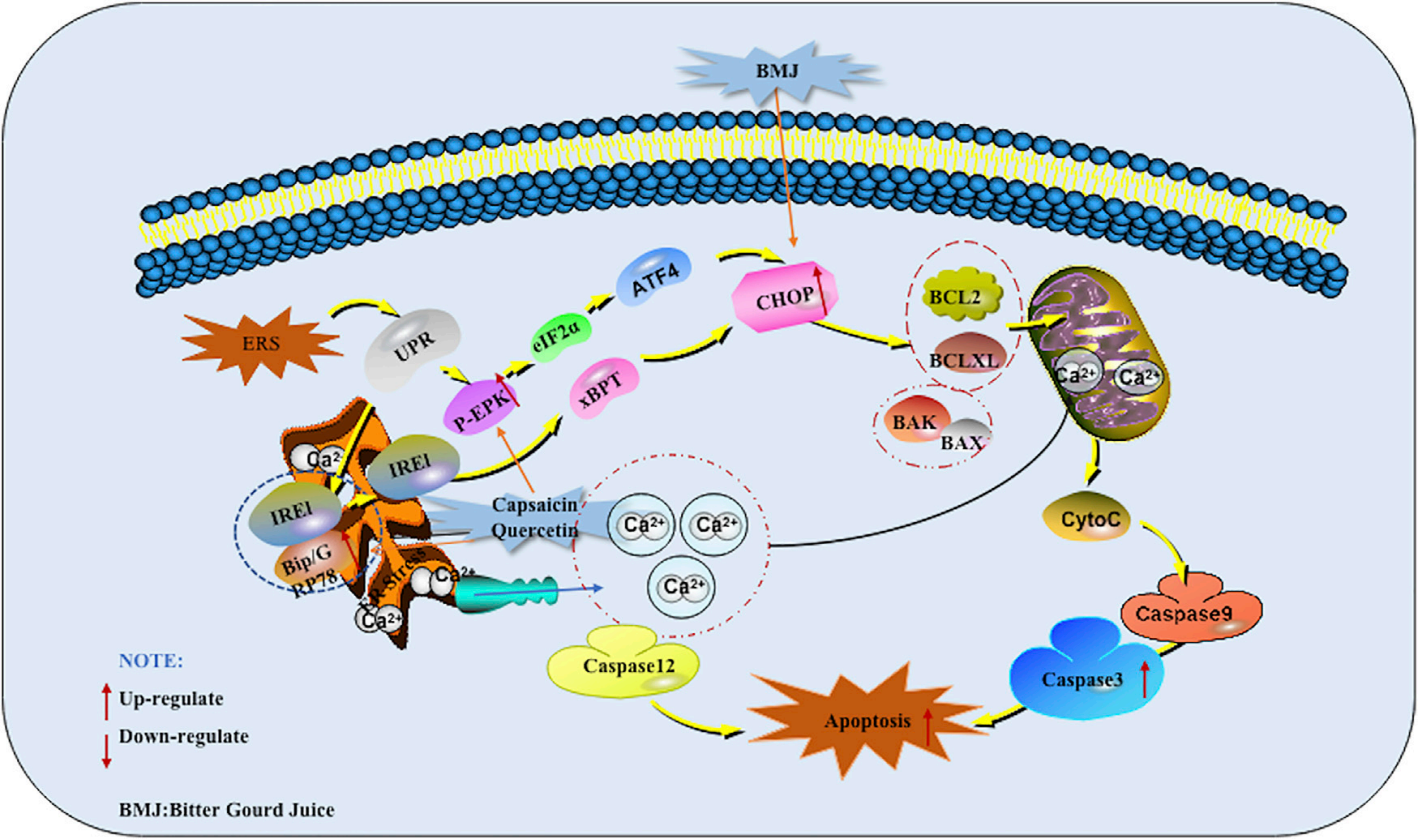
Endoplasmic reticulum stress (ERS) mediated apoptotic pathway of pancreatic cancer induced by herbal medicines.

In 2013, Lin et al. found that Capsaicin can significantly enhance the expression of GRP78 in PANC-1 and SW1990 cells. At the same time, GADD153, a marker of ERS mediated apoptotic pathway, was also added. Further research found that Capsaicin prolonged the survival rate of nude mice with orthotopic pancreatic cancer xenograft tumors.The results of western blot analysis showed that the protein expression of GRP78, phospho-PERK, phospho-eIF2α, ATF4 (eIF2 α downstream target), and GADD153 was much higher in the tumor tissues of Capsaicin-treated mice compared with that of control group. This study is the first to study the effect of Capsaicin on endoplasmic reticulum-mediated apoptosis in pancreatic cancer *in vitro* and *in vivo* (Lin et al., 2013). Kaur M et al. also found an increase in CHOP levels when studying the mechanism of BMJ induced apoptosis in 2013, so the induction of CHOP levels by BMJ may also help induce apoptosis (Kaur et al., 2013). In 2013, Lee et al. investigated the mechanism of Quercetin inducing apoptosis in pancreatic cancer, found that Quercetin went through the ERS mediated apoptotic pathway which induced the increase of GADD153/CHOP protein expression finally by increasing Grp78/Bip protein as well as activating PERK protein in PANC-1 cells. At the same time, it was also found that intracellular calcium accumulation in cell (Lee et al., 2013). The potential mechanisms of herbal medicine for inducing apoptosis in this part are summarized in Figure 3 and Table 1.

### PI3K-Akt Mediated Apoptotic Pathway

**FIGURE 4:**
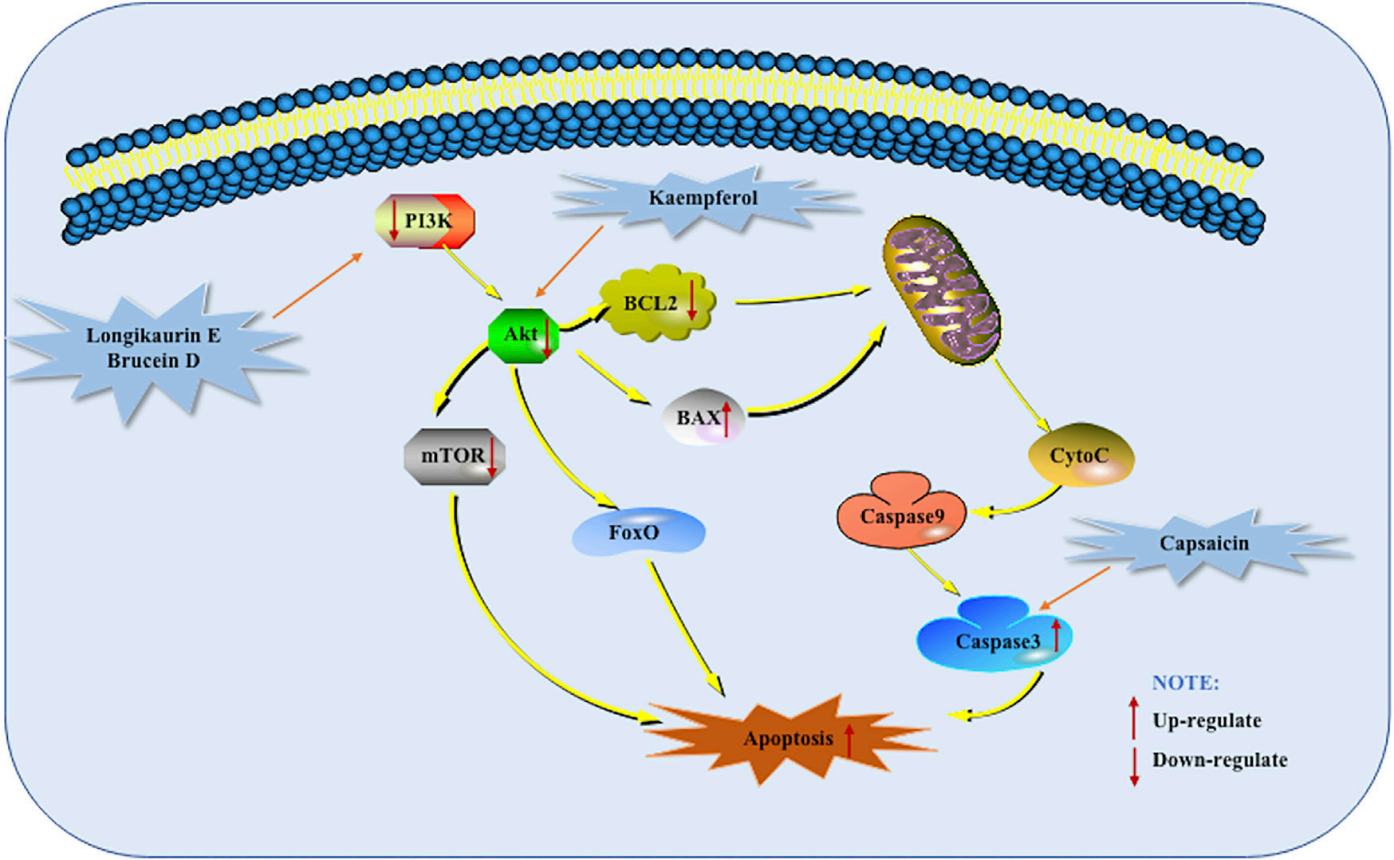
PI3K-Akt mediated apoptotic pathway of pancreatic cancer induced by herbal medicines.

PI3K constitutes a family of lipid kinases whose role is that they can phosphorylate inositol ring 3′-OH group in inositol phospholipids to generate the second messenger phosphatidylinositol-3,4,5-triphosphate (PI -3,4,5 -P (3)). RPTK activation causes PI3K inside the plasma membrane to produce PI (3,4,5) P 3) and PI (3,4) P (2). Akt interacts with these phospholipids, causing its translocation to the inner membrane, which is phosphorylated and activated by PDK1 and PDK2. Activated Akt regulates the functions of a variety of substrates. These substrates are involved in regulating cell survival, cell cycle progression and cell growth. It has been shown that the components of the PI3K-Akt Mediated Apoptotic Pathway are often altered and inappropriately activated in human cancers (Fresno Vara et al., 2004). Bondar et al. studied 9 human pancreatic cancer cell lines *in vitro* and observed that onstitutive AKT phosphorylation (on S473) was consistent with pathway activation in seven of nine human pancreatic carcinoma cell lines *in vitro*. This study determined that the PI3K-Akt Mediated Apoptotic Pathway is constitutively activated in most human pancreatic cancer cell lines, and determined that this pathway is a promising target for therapeutic intervention (Bondar et al., 2002). Therefore, the PI3K-Akt Mediated Apoptotic Pathway can be considered as a feasible and effective target for pancreatic cancer treatment. In 2013, Zhang et al. did a corresponding study on Capsaicin. In mice treated with Capsaicin, the expression of Tyr458 and Ser473 was down-regulated, and the expression of Caspase-3 was promoted. This indicates that the down-regulation of the PI3K-Akt mediated apoptotic pathway may be related to the apoptosis of PANC-1 cells induced by Capsaicin. The same result was found when conducting *in vitro* research experiments (Zhang et al., 2013). When Cheng et al. studied Longikaurin E in 2015, they found that p38 phosphorylation increased and decreased phosphorylation of PI3K/AKT pathway (Cheng et al., 2015). In 2017, Lai et al. studied the inhibitory effect of Brucein D on pancreatic cancer and found that Brucein D can induce PANC-1 and Capan-2 cells apoptosis through ROS-Associated PI3K-Akt mediated apoptotic pathway. Further research found Brucein D Suppresses the Tumor Progression in Orthotopic Xenograft Mouse Model. The protein expression of phosphorylated forms of Akt (Ser473) and Akt (Thr308) proteins was drastically suppressed by Brucein D treatment. These data suggested that modulation of PI3K/Akt activity might be an important molecular mechanism underlying the *in vivo* anti-PanCa effects exerted by Brucein D (Lai et al., 2017). MarElia et al. found in 2018 that the use of Anemarrhena and Anemarrhena saponins alone or together with Gem can inhibit the phosphorylation of PI3K-Akt Mediated Apoptotic Pathway proteins. Anemarrhena and Anemarrhena saponins enhance the effect of Gem in a dose-dependent manner. In addition, this study also found that Anemarrhena and Anemarrhena saponins can promote PANC-1 cell apoptosis through a Caspase-dependent apoptosis mechanism (MarElia et al., 2018). TGM2 is a multifunctional protein. It is the first mammalian transglutaminases (TGs) member to be discovered and is involved in the pathogenesis of many cancers. Recently. Wang F et al. found that Kaempferol increases ROS levels, which in turn suppresses the Akt/mTOR signaling pathway. At the same time, the down-regulation of TGM2 was also detected. *In vivo* research also found that Kaempferol promotes apoptosis via the TGM2-mediated ROS-dependent Akt/mTOR signaling pathway. TGM2 may be a potential target of kaempferol to inhibit pancreatic cancer and could serve as a promising prognostic biomarker for this disease (Wang J. W. et al., 2021). The potential mechanisms of herbal medicine for inducing apoptosis in this part are summarized in Figure 4 and Table 1.

### NF-κB Mediated Apoptotic Pathways

The transcription factor NF-κB is a major cell regulatory factor, as well as a chemical activator, growth factor, and cell adhesion molecule. NF-κB binds to its inhibitory protein IκB and is located in the cytoplasm. They are not active in normal cells. Once IκB is stimulated outside the cell, IκB is phosphorylated and degraded, so that NF-κB is activated and migrates to the nucleus, and then start or enhance the transcription of related genes. Cellular activities related to NF-κB include cell connection and repair, inflammatory cell displacement, early disease-causing signal amplification and spread, and tumor occurrence and development (Jin., 2013; Chen et al., 2020). At present, NF-κB Mediated Apoptotic Pathways has attracted more and more attention. Modern pharmacological studies have proved that Chinese herbal medicine can induce apoptosis of pancreatic cancer cells by regulating the NF-κB Mediated Apoptotic pathway.

**FIGURE 5:**
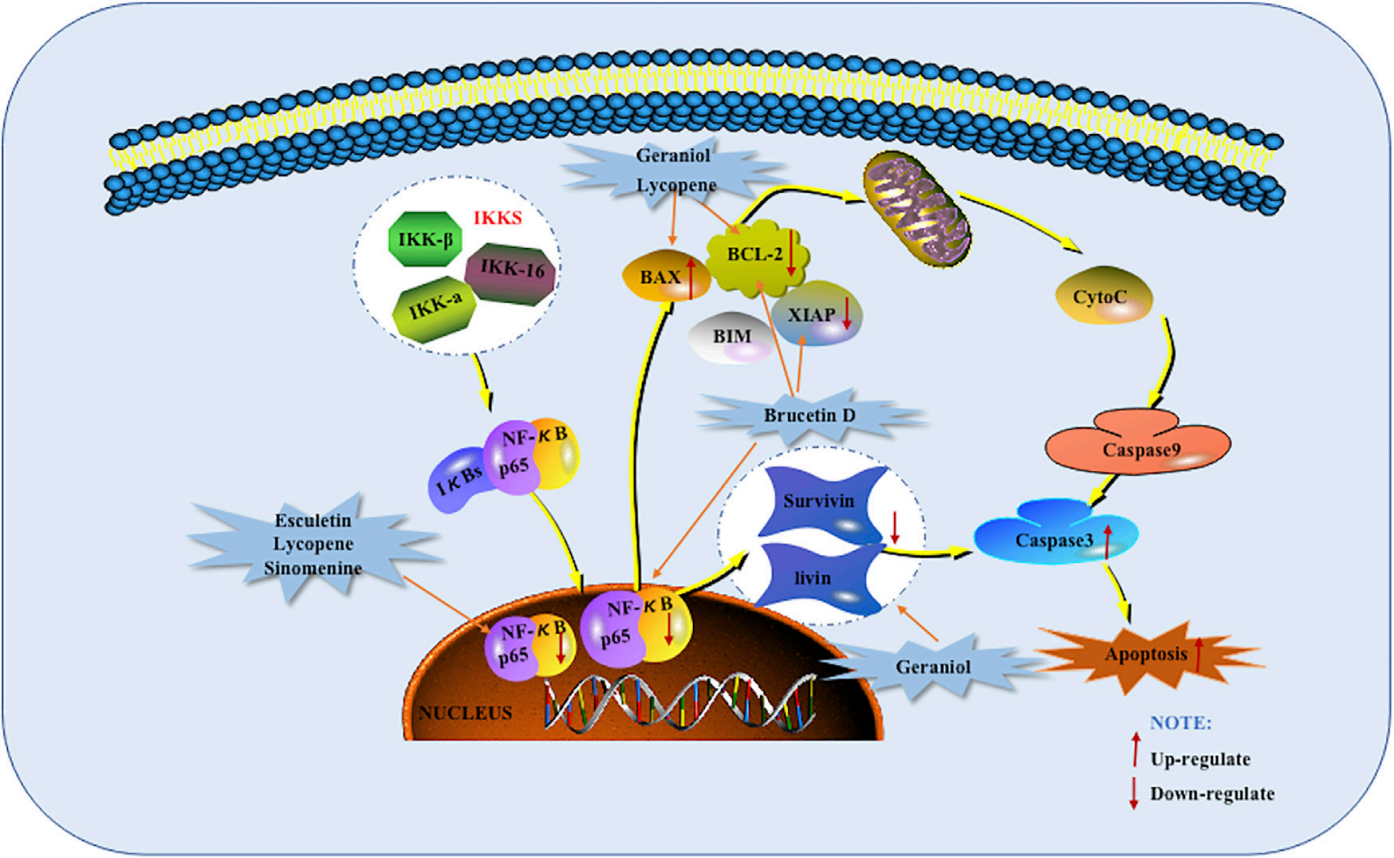
NF-κB mediated apoptotic pathway of pancreatic cancer induced by herbal medicines.

As early as 2010, Lau ST and others found that Brucetin D can inhibit the anti-apoptotic activity of NF-κB and promote apoptosis. Brucetin D treatment also weaken the p65 activation in PANC-1 cells, resulting in a decrease in the transcription of Bcl-2 and XIAP. Also found that Brucetin D inhibits the growth of CAPAN-2 human pancreatic tumor xenografts *in vivo* (Lau et al., 2010). Livin and Survivin, which are located in the nucleus of the apoptosis protein inhibitor family, are two downstream genes of NF-κB. They can inhibit cell apoptosis by inhibiting the expression of Caspase. Studies have shown that the two are not expressed or low expressed in normal pancreatic tissues, while they are highly expressed in pancreatic cancer tissues. In 2013, when Jin was researching Geraniol, she found that Geraniol inhibited the expression of Livin and Survivin, and might thereby enhance the sensitivity of pancreatic cancer to Gem. NF-κB is a regulatory factor for the promoter of the important apoptosis inhibitor protein Bcl-2. Due to the abnormal increase of NF-κB activity in pancreatic cancer, which leads to a large number of activation of the Bcl-2 promoter, eventually leading to the acceleration of cancer cell proliferation and the inhibition of apoptosis. This study also found that Geraniol inhibited the binding ability of NF-κB and the promoter. At the same time, Geraniol can also down-regulate the expression of the suppressor gene Bcl-2 and up-regulate the expression of the pro-apoptotic Bax gene, thereby promoting tumor cell apoptosis (Jin., 2013). In 2016, Arora et al. found that Esculetin binds to Keap1 and inhibits its interaction with Nrf2 in pancreatic cancer cells. Therefore, the nuclear accumulation of Nrf2 in PANC-1 cells is promoted. Nrf2 is phosphorylated and transported to the nucleus, where it binds to the promoter with the ARE sequence to eliminate ROS and weaken NF-κB to induce anti-proliferation and apoptosis (Arora et al., 2016). When studying Lycopene in 2019, Jeong et al. found that it inhibits NF-κB activation and the expression of NF-κB target genes such as cIAP1, cIAP2 and survivin by reducing ROS levels. The NF-κB target genes (cIAP1, cIAP2 and survivin) inhibit Caspase-3, which means that Lycopene promotes the activation of Caspase-3. Therefore, Lycopene induces Caspase-3 dependent apoptosis and increases the ratio of Bax to Bcl-2 in PANC-1 cells (Jeong et al., 2019). In 2020, Chen et al. found that Sinomenine can inhibit the activation of NF-κB mediated apoptotic pathways, down-regulate the expression of tumor cell anti-apoptotic factors and apoptosis inhibitor proteins, promote the lysis of Caspase-3, and promote pancreatic cancer Capan-1 apoptosis (Chen et al., 2020). The potential mechanisms of herbal medicine for inducing apoptosis in this part are summarized in Figure 5 and Table 1.

### MAPK Mediated Apoptotic Pathway

**FIGURE 6:**
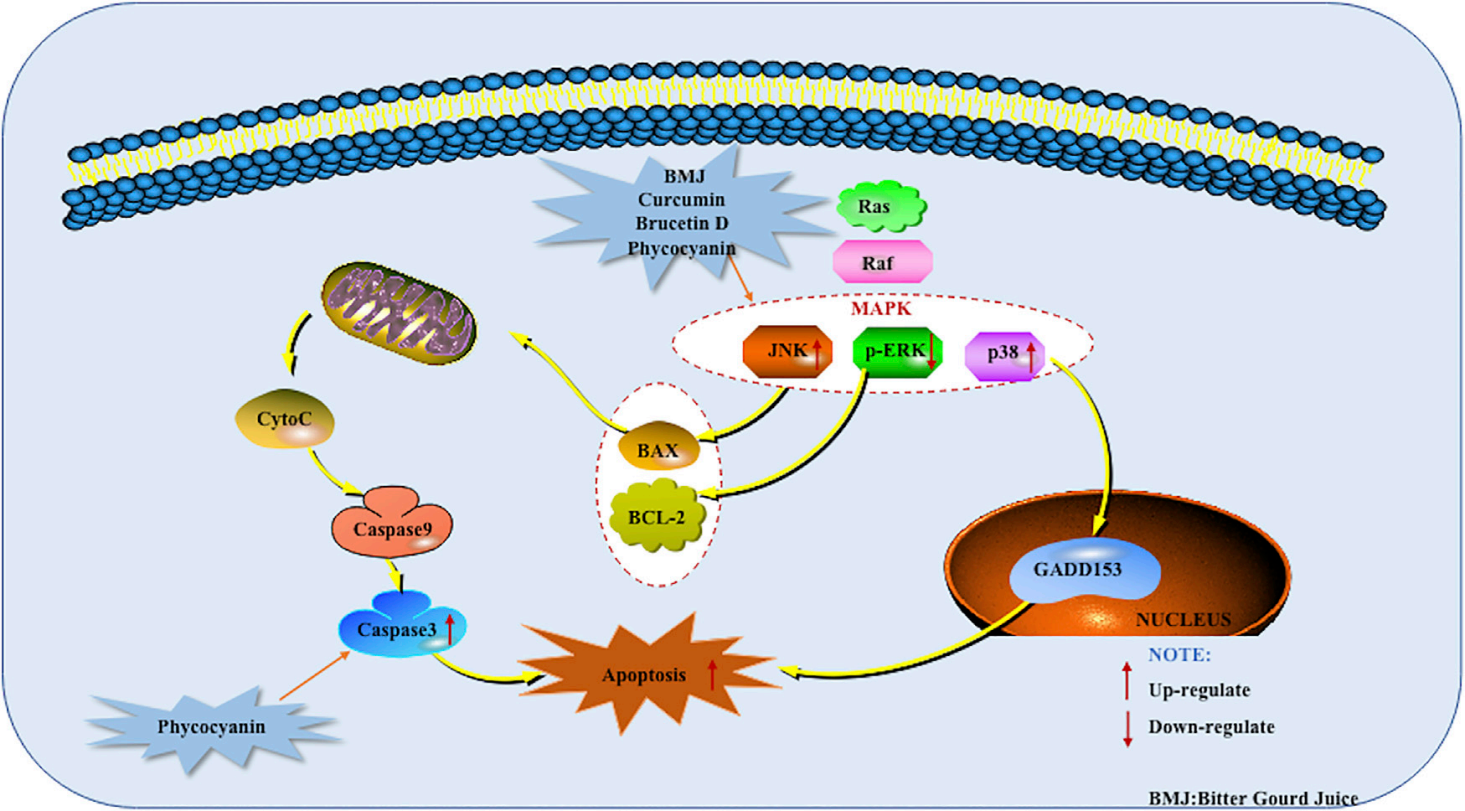
Mitogen-activated protein kinase (MAPK) mediated apoptotic pathway of pancreatic cancer induced by herbal medicines.

Mitogen-activated protein kinase (MAPK) is a silk protein/threonine kinase, an important molecule that transmits eukaryotic signals. MAPK is involved in the regulation of gene expression, cell proliferation and death, and plays a key role in the signal transmission process of multiple receptors. MAPKs mainly include extra cellular signal-regulated protein kinases (ERK), P38 mitogen activated protein kinase (p38MARK), and c-Jun N-terminal kinase (JNK). Among them, the p38MAPK plays an important role in the stress response such as inflammation and apoptosis. A variety of inflammatory factors, growth factors, and stress response will double phosphorylate the tyrosine and threonine of p38 protein, thereby activating p38. The activated p38 can enter the nucleus or transfer to other parts to activate transcription factors. Such as ATF2/6, ATH-1/2, ETS-1, MAX, HSF-1, nuclear transcription factor β, SAP-1, etc. These transcription factors in turn regulate the cytokines involved in cellular responses such as TNF-α, IL-1, IL-6, IL-8, and so on (Xu, 2019). When the cell is stimulated by the external environment, the c-Jun N-terminal kinase will be activated, and a part of the activated JNK will be translocated to the nucleus, and form p-JNK after phosphorylation modification. P-JNK can act on the pro-apoptotic proteins Bax and Bak in the Bcl family to induce the release of Cyt-C into the cytoplasm, thereby initiating the apoptosis program. It is reported that MAPK plays a vital role in inducing pancreatic cancer cells. In recent years, the influence of Chinese herbal medicine and its active monomers on MAPK Mediated Apoptotic Pathway in pancreatic cancer has been comprehensively reported *in vivo* and *in vitro*. When studying Curcumin in 2006, Lev-Ari S et al. found that it promotes the increase of apoptosis by inhibiting the activity of the ERK1/2 signaling pathway, and has a particularly strong effect on pancreatic cancer cell lines that express COX-2 (Lev-Ari et al., 2006). In 2010, Lau et al. found that Brucetin D mediates the activation of ROS to regulate the p38 signaling pathway to induce cell apoptosis. *In vivo* experiments found Brucetin D inhibits the growth of CAPAN-2 human pancreatic tumor xenografts (Lau et al., 2010). Later, in 2019, Huang et al. found that Brucein D combined with paclitaxel can also induce apoptosis of Capan-2 cells by activating JNK phosphorylation (Huang et al., 2019). When studying BMJ in 2013, Kaur et al. also found that the treatment of BMJ resulted in the prolongation and continuous activation of p38 and ERK1/2. Therefore, activation of p38 and ERK1/2 may also help induce apoptosis. Further use of IHC analysis found that BMJ’s in vivo efficacy against MiaPaCa-2 xenograft growth is through inhibiting proliferation, inducing apoptosis and activating AMPK (Kaur et al., 2013). In 2016, Liao et al. found that the natural product Phycocyanin (from Spirulina) induced cell death in PANC-1 cells only partially dependent on the activation of Caspase-3. The activation of the JNK and p38 pathways while inhibiting ERK signaling indicates that the MAPK signaling pathway plays a key role in the apoptosis of cancer cells induced by Phycocyanin (Liao et al., 2016). The potential mechanisms of herbal medicine for inducing apoptosis in this part are summarized in Figure 6 and Table 1.

### Other Reported Pathways

MiRNAs/miRs are a class of small non-coding RNAs, and miRNAs play an important role in cytogenetics. In the process of tumor occurrence and development, it is related to the proliferation, apoptosis, differentiation, and metastasis of tumor cells. Therefore, miRNA has become a promising drug target. In 2014, Ma et al. found that curcumin inhibits cell growth, migration, and invasion, and induces apoptosis, which is related to the increased expression of miR-7 and the subsequent decrease in the expression of SET8 (one of the miR-7 targets) (Ma et al., 2014). Then in 2017, Yang et al. found that curcumin treatment significantly induced the expression of miR-340. In addition, an anti-apoptotic protein called XIAP was targeted by miR-340, leading to the pro-apoptotic effect of curcumin. Therefore, this study shows that the miR-340/XIAP signaling pathway is essential for curcumin-induced apoptosis of pancreatic cancer cells (Yang et al., 2017). In 2020, Wang et al. found that Honokiol induced the up-regulation of miR-101 in pancreatic cancer cells. Overexpression of miR-101 reduced cell viability and increased the activity of Caspase-3 and the rate of apoptosis. It also inhibited cell invasion, and it was also found that miR-101 down-regulated the expression of Mcl-1. As a downstream target of miR-101, Mcl-1 exerts an anti-apoptotic function. Further studies have found that Honokiol suppressed *in vivo* tumor growth of PANC-1 cells in a dose-dependent manner. It was also found that Honokiol dose-dependently up-regulated the expression of miR-101 and down-regulated the expression of Mcl-1, which is consistent with *in vitro* studies. (Wang et al., 2020). In 2021, Huang et al. found that Icariin can inhibit the proliferation of pancreatic cancer BxPC-3 cells and induce apoptosis of BxPC-3 cells by up-regulating miR-9 (Huang et al., 2021).

In addition, natural products also have other induction mechanisms. As early as 2007, Phillips et al. found that Triptolide induces the death of pancreatic cancer cells by inhibiting the expression of Hsp70. *in vivo* studies have also confirmed this (Phillips et al., 2007). In the same year, Zhou et al. found that the apoptosis of pancreatic tumor cell lines induced by Triptolide is related to the inhibition of 5-LOX (Zhou et al., 2007) ASK1 is a member of the mitogen-activated protein kinase family and is mainly activated by ROS. Previous studies have shown that ASK1 plays a key role in oxidative stress-mediated apoptosis by activating JNK and p38 signaling pathways. In 2012, Pramanik et al. found that Capsaicin can disrupt the Trx-ASK1 interaction through ROS, thereby inhibiting Trx to activate ASK1, leading to pancreatic tumor cell apoptosis. Further research found that Capsaicin suppresses AsPC-1 tumor growth *in vivo* in athymic nude mice. Capsaicin treatment also observed cleavage caspase-3 and PARP, inhibits Trx and activates ASK1 in pancreatic tumors (Pramanik and Srivastava, 2012). In 2019, Guo et al. found that diosgenin-induced apoptosis in pancreatic cancer cells may be related to down-regulation of EZH2 (oncoprotein). They also observed that administration of Diosgenin clearly inhibited subcutaneous tumor growth of nude mice compared with control group (Guo et al., 2019). Clinical studies have found that IL-6, JAK2, STAT3, etc. are abnormally expressed in tumor tissues of patients with pancreatic cancer. IL-6 is an important indicator for judging the prognosis of malignant tumors. After IL-6 binds to the corresponding IL-6 receptor on the target cell, it activates glycoprotein 130 (GP130) on the cell membrane surface, thereby activating JAK associated with GP130, thereby promoting the activation of receptor protein tyrosine kinases and binding to STAT3. Then the nuclear factor NF-κB is induced to dissociate from the IκB complex into free phosphorylation, and then enter the nucleus through the nuclear membrane. NF-κB binds to its corresponding DNA response elements to regulate the expression of inflammation, apoptosis, or the proliferation of related factors. In 2020, Meng et al. found that Gentiopicroside can inhibit the proliferation and induce apoptosis of pancreatic cancer cells PANC-1. The mechanism may be related to the inhibition of the IL-6/JAK2/STAT 3 signaling pathway (Meng et al., 2020). In the same year, Hu et al. found that Polyphyllin VII induced Miapaca-2 cell apoptosis in a dose-dependent manner (Hu et al., 2020). Recently, He et al. found that Polyphyllin VII may induce PANC-1 cell apoptosis by down-regulating the expression of PD-L1 (He et al., 2021).

## Conclusions

**FIGURE 7:**
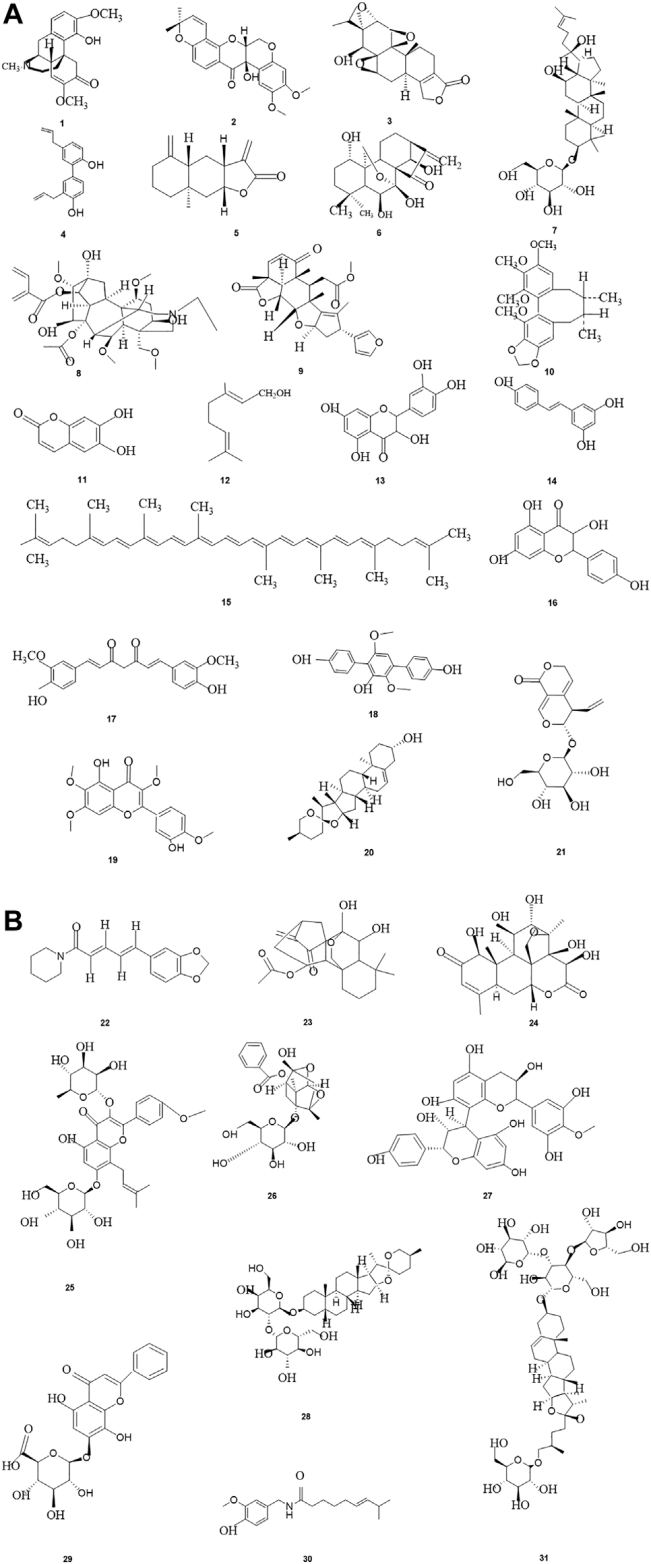
**(A–B)** The related monomers of inducing apoptosis in Pancreatic cancer.Sinomenine (1), Tephrosin(2),Triptolide(3), Honokiol(4), Isoalantolactone(5), Oridonin(6), Ginsenosid-e Rh2(7), Aconitine(8), Nimbolide(9), Schisandrin B(10), Esculetin(11), Geraniol(12), Quercetin(13), Resveratrol(14), Lycopene(15), Kaempferol(16), Curcumin(17), Terphenyllin(18), Casticin(19), Diosgenin(20), Gentiopicroside(21), Piperine(22), Longikaurin E(23), Brucein D(24),Icariin(25), Paeoniflorin(26), Proanthocyanidins(27), Timosaponin-AIII(28), Glychionide-A(29), Capsaicin (30), Polyphyllin VII(31).

**FIGURE 8:**
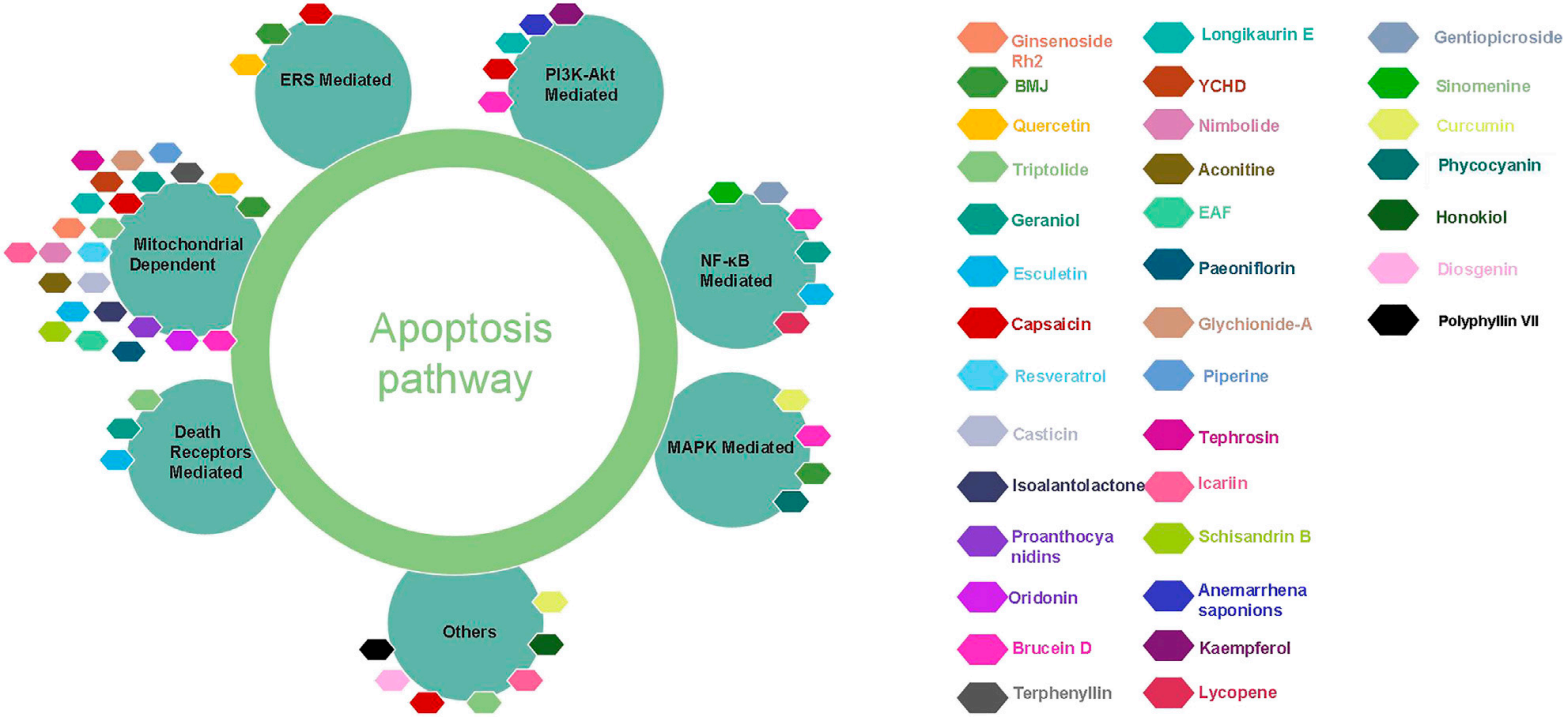
The different apoptotic pathways of the natural monomers isolated from herbal medicines. YCHD, Yin Chen Hao Decoction; BMJ, Bitter Gourd Juice.

From ancient times to the present, herbal medicines play important roles in maintaining human health and curing diseases (Newman and Cragg, 2020; Zhang et al., 2021), and are generally regarded as valuable resources for screening and searching for new drug candidates with less toxicity. Pancreatic cancer, one of the most lethal malignancies and called the King of cancer, is the fourth leading cause of cancer-related death worldwide now, with an estimated 5-year survival rate less than 10%. Apoptosis, as the most typical way of programmed cell death and physiological cell suicide, is increasingly regarded as an ideal way to treat cancer. Increasing studies have found that herbal medicines and their monomers have pro-apoptotic effects on pancreatic cancer cells, we have summarized them in this review. The important mechanisms of herbal medicines in inducing apoptosis of pancreatic cancer cells include death receptors mediated apoptotic pathway, mitochondrial dependent apoptotic pathway, NF-κB mediated apoptotic pathways, MAPK mediated apoptotic pathway, ERS mediated apoptotic pathway, PI3K-Akt mediated apoptotic pathway, and other reported pathways such as JAK-STAT signal pathway. The classification of compounds that induce apoptosis in pancreatic cancer cells is shown in Table 2, the compound structure diagram is shown in Figure 7A,B, and the distribution diagram of compounds with different induction mechanisms is shown in Figure 8.

**TABLE 2:** Monomers for inducing apoptosis of pancreatic cancer.

Classification	Monomers	Apoptotic pathways	References
Terpenoids	Triptolide	Mitochondrial dependent apoptosis, Death Receptors mediated apoptosis, Inhibit Hsp70, 5-LOX	Chen et al. (2013), Wang et al. (2012), Phillips et al. (2007), Zhou et al. (2007)
	Geraniol	Death Receptors mediated apoptosis, Mitochondrial Dependent apoptosis, NF-κB mediated apoptosis	Jin. (2013)
	Oridonin	Mitochondrial dependent apoptosis	Qi et al. (2012)
	Brucein D	Mitochondrial dependent apoptosis, NF-κB mediated apoptosis, MAPK mediated apoptosis, PI3K-Akt mediated apoptosis	Huang et al. (2019), Lau et al. (2010), Lai et al. (2017)
	Lycopene	NF-κB mediated apoptosis	Jeong et al. (2019)
	Nimbolide	Mitochondrial dependent apoptosis	Subramani et al. (2016)
	Isoalantolactone	Mitochondrial dependent apoptosis	Ding. (2012)
	Longikaurin E	Mitochondrial dependent apoptosis	Cheng et al. (2015)
Alkaloids	Aconitine	Mitochondrial dependent apoptosis	Ji et al. (2016)
	Piperine	Mitochondrial dependent apoptosis	Zhong et al. (2020)
	Sinomenine	NF-κB mediated apoptosis	Chen et al. (2020)
Flavonoids	Casticin	Mitochondrial dependent apoptosis	Ding. (2012)
	Proanthocyanidins	Mitochondrial dependent apoptosis	Prasad et al. (2012)
	Quercetin	Mitochondrial dependent apoptosis, ERS mediated apoptosis	Lee et al. (2013)
	Icariin	Mitochondrial dependent apoptosis, Up-regulate miR-9	Huang et al. (2021)
	Kaempferol	PI3K-Akt-mTOR mediated apoptosis	Wang et al. (2021a)
	Tephrosin	Mitochondrial dependent apoptosis	Du et al. (2021)
Steroids	Ginsenoside Rh2	Mitochondrial dependent apoptosis	Tang et al. (2013)
	Diosgenin	Down-regulate EZH2	Guo et al. (2019)
	Timosaponin-AIII	PI3K-Akt mediated apoptosis	MarElia et al. (2018)
Lignans	Schisandrin B	Mitochondrial dependent apoptosis	Wang et al. (2021b)
	Honokiol	Up-regulate miR-101	Wang et al. (2020)
Coumarin	Esculetin	Mitochondrial dependent apoptosis, Death Receptors mediated apoptosis, NF-κB mediated apoptosis	Arora et al. (2016)
Phenols	Resveratrol	Mitochondrial dependent apoptosis	Roy et al. (2011), Duan et al. (2016)
	Terphenyllin	Mitochondrial dependent apoptosis	Zhang et al. (2020)
	Capsaicin	Mitochondrial dependent apoptosis, PI3K-Akt mediated apoptosis, ERS mediated apoptosis, Inhibit Trx, activate ASK1	Zhang et al. (2008), Zhang et al. (2013), Lin et al. (2013), Pramanik and Srivastava, (2012)
	Curcumin	MAPK mediated apoptosis, Increase miR-7, decrease SET8, miR-340/XIAP signal pathway	Lev-Ari et al. (2006), Ma et al. (2014), Yang et al. (2017)
Glucosides	Paeoniflorin	Mitochondrial dependent apoptosis	Li et al. (2017)
	Glychionide-A	Mitochondrial dependent apoptosis	Yu et al. (2019)
	Polyphyllin VII	Down-regulate PD-L1	He et al. (2021)
	Gentiopicroside	IL-6/JAK2/STAT3 signaling pathway	Meng et al. (2020)
Other	Phycocyanin	MAPK mediated apoptosis	Liao et al. (2016)

From the literature searched, mitochondrial dependent apoptosis is the most reported signaling pathway for herbal medicine to induce apoptosis in pancreatic cancer cells. For example, proanthocyanidins induced cells apoptosis through down-regulation of Bcl-2 and Bcl-xL, up-regulation of Bax and activate Caspase-3. Brucein D induced cells apoptosis via multi-pathways by mitochondrial dependent apoptotic pathway, down-regulate PI3K/AKT signaling, inhibit NF-κB, up-regulate p38 to activate MAPK mediated apoptotic pathway, so Brucetin D is one of the most promising active ingredients, but clinical research needs to be strengthened. Understanding that herbal medicine induces apoptosis of pancreatic cancer cells will not only help clarify the molecular mechanism of herbal medicine in treating diseases, but also help to find new drug candidates. Therefore, this review highlights the molecular mechanisms by which herbal medicines and their components induce apoptosis in pancreatic cancer cells, and provides some directions for the clinical future development of these herbal medicines against pancreatic cancer.
